# An optical system via liquid crystal photonic devices for photobiomodulation

**DOI:** 10.1038/s41598-018-22634-w

**Published:** 2018-03-09

**Authors:** Chia-Ming Chang, Yi-Hsin Lin, Abhishek Kumar Srivastava, Vladimir Grigorievich Chigrinov

**Affiliations:** 1 National Chiao Tung University, Department of Photonics, Hsinchu, 30010 Taiwan Republic of China; 2 Hong Kong University of Science and Technology, Department of Electronic and Computer Engineering, Hong Kong, China

## Abstract

Photobiomodulation or low-level light therapy (LLLT) has extensive applications based on light-induced effects in biological systems. Photobiomodulation remains controversial because of a poorly understood biochemical mechanism limited by the well-known biphasic dose response or Arndt-Schulz curve. The Arndt-Schulz curve states that an optimal dose of light is a key factor for realizing a therapeutic effect. In this report, we demonstrate a tunable optical system for photobiomodulation to aid physicians in overcoming the constraints of light due to biphasic dose response. The tunable optical system is based on a white light-emitting diode and four liquid crystal (LC) photonic devices: three LC phase retarders, and one LC lens. The output light of the tunable optical system exhibits electrical tunability for the wavelength, energy density and beam size. The operating principle is introduced, and the experimental results are presented. The proposed concept can be further extended to other electrically tunable photonic devices for different clinical purposes for photobiomodulation.

## Introduction

Photobiomodulation, also known as low-level light therapy (LLLT), was first demonstrated to aid hair growth in mice in 1968^1,2^. Clinical studies suggest that light provided by either lasers or light-emitting diodes (LED) with a wavelength in the range of 600–1100 nm at a fluence of <10 J/cm^2^ and with an intensity (or irradiance) of 3–90 mW/cm^2^ can not only promote hair growth, but also assist in wound healing, pain reduction, tissue repair, anti-inflammatory therapy, traumatic brain injury, spinal cord injury, depression, Parkinson’s disease, and circadian rhythm sleep disorder^3–14^. Moreover, the use of blue light and green light opens up opportunities to exploit stem cells in regenerative medicine^15–19^. Red light can even be used to effectively facilitate the motility of spermatozoa^20^. Stimulation by visible light accomplished with virtual images provides novel therapies in neuroscience^21,22^. Many studies also reveal the benefits of LLLT in health, such as the enhancement of memory, attention, and executive functions^23^. However, LLLT remains controversial because of poor understanding of the underlying biochemical mechanism as well as the selection of appropriate dosimetric parameters, such as wavelength, fluence (or energy density in units of Joule/cm^2^), power density, and pulse duration for the applied light^24–26^. The optimal dose of light is particularly important in LLLT because of the biphasic dose response or the Arndt-Schulz curve, which states that the therapeutic effect depends on the light dose^27–30^. When the energy density is either too weak or too strong, light exposure results in either no observable effect or inhibition of cellular function. The irradiance threshold is also a basic factor for extensive applications in photobiomodulation^31^. An optimal dose of light is necessary to reduce negative therapeutic results. However, the adjustment of parameters relating to light in clinical studies is less flexible for physicians. This motivates us to develop electrically tunable light modulators for light sources, which can enable researchers and physicians to adjust light parameters easily to study the biochemical mechanism of photobiomodulation. Since liquid crystals (LCs) are superior in the modulation of light amplitude and optical phase, versatile photonic devices have been designed based on LC materials, such as attenuators, lenses, waveplates (wave retarders), gratings, polarization rotators and color filters^32–43^. LC photonic devices show the advantages of being electrically tunable, lightweight and portable. To date, no study has been reported for the design of tunable optical systems or LC photonic devices for photobiomodulation. In this paper, we demonstrate a tunable optical system for photobiomodulation to aid physicians in overcoming the constraints of light use due to the biphasic dose response. The output light in the optical system we proposed exhibits electrical tunability for the wavelength, energy density and beam size. In addition to a white light-emitting diode (LED), the optical system mainly consists of four LC devices: three LC phase retarders and one LC lens. We start from the operating principle to explain how to design an optical system based on LC devices to realize electric tunability for the selection of wavelength and fluence. Thereafter, lab-fabricated LC phase retarders and an LC lens are adapted to demonstrate the concept. The concept in the paper can be further extended to other electrically tunable photonic devices that are tailored for different clinical purposes in photobiomodulation.

## Operating Principle

**Figure 1:**
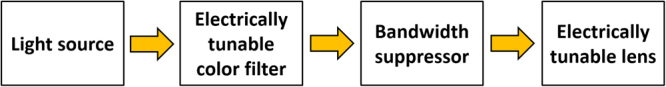
Four parts of a tunable optical system for LLLT.

**Figure 2:**
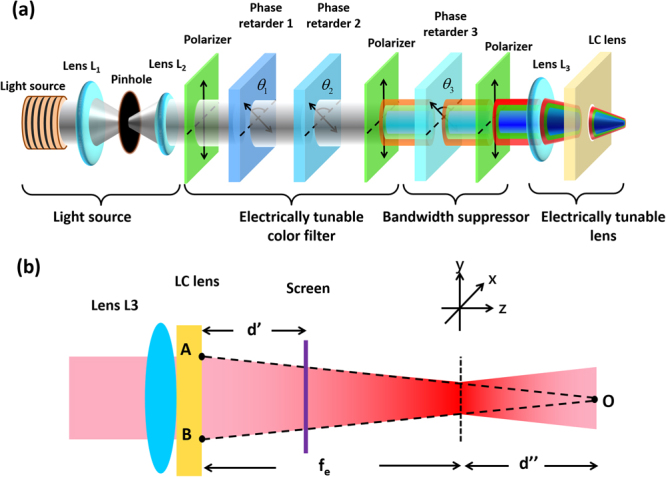
(**a**) The structure of the proposed optical system for portable LLLT. (**b**) Detailed beam propagation in lens L_3_ and the LC lens.

To harness the fluence (in a unit of J/cm^2^) and select a wavelength of light, the basic tunable optical system for LLLT should consist of four parts, namely, a light source, an electrically tunable color filter, a bandwidth suppressor and an electrically tunable lens, as shown in Fig. 1. The function of the electrically tunable color filter is to select the required wavelength for LLLT applications. The bandwidth suppressor narrows the bandwidth of the color of light selected by the electrically tunable color filter. In addition to wavelength, adjustment of the beam size is also required. The function of the electrically tunable lens is to adjust the beam size, which results in adjustment of the light intensity (in units of W/cm^2^). By controlling the period of the switching time for the light source, the exposure time can be manipulated, and thus, the fluence can also be adjusted. To further realize the concept shown in Fig. 1 using LC devices, the detail design is illustrated in Fig. 2. The white light source is an LED device (TouchBrigh TB-X3-RCPI). Two lenses (L_1_ and L_2_) and a pinhole are used for the collimation of light. The lens powers of the solid lenses L_1_ and L_2_ are 20 and 50 diopter (or m^−1^), respectively. Electrically tunable color filter consists of two polarizers and two electrically tunable LC phase retarders (i.e., Phase retarder 1 and Phase retarder 2). The transmissive axes of the two polarizers are parallel to the y-direction, and the initial alignment of the two phase retarders is either 135° or 90° with respect to the x-axis. The effective slow axis of the phase retarders can be switched along * θ* = 135° at +10 V or *θ* = 90° at −10 V (i.e.,*θ*_1_ and *θ*_2_ in Fig. 2 are 135° or 90° depending on the applied voltage) due to the bistability of ferroelectric LC (FLC). The bandwidth suppressor consists of another LC phase retarder (i.e., Phase retarder 3) and a polarizer. The electrically tunable lens consists of a lens (L_3_) with a lens power of 12 D and an LC lens with electrically tunable lens power.

The Jones calculus is adopted to verify the structure shown in Fig. 2. When *θ*_*i*_ = 135° or 90°, the Jones matrices (*P*_*i*_) for the LC phase retarders are expressed as^33^:

1 $${P}_{i}({\theta }_{i}={135}^{\circ })=\frac{1}{2}[\begin{array}{cc}1+{e}^{j{{\rm{\Gamma }}}_{i}} & 1-{e}^{j{{\rm{\Gamma }}}_{i}}\\ 1-{e}^{j{{\rm{\Gamma }}}_{i}} & 1+{e}^{j{{\rm{\Gamma }}}_{i}}\end{array}]\,;i=1,2$$

2 $${P}_{i}({\theta }_{i}={90}^{\circ })=[\begin{array}{cc}1 & 0\\ 0 & {e}^{j{{\rm{\Gamma }}}_{i}}\end{array}]\,;i=1,2$$

where *i* = 1 or 2 represents phase retarder 1 or phase retarder 2, *θ* is the angle between the slow axis and x-axis, and Γ_*i*_ is the phase retardation, which satisfies the relation ${{\rm{\Gamma }}}_{i}=2\pi \cdot {\rm{\Delta }}{n}_{i}\cdot {d}_{i}/\lambda$, where *λ* is the wavelength, Δ*n*_*i*_ is the birefringence of the LC (here, ${\rm{\Delta }}{n}_{1}={\rm{\Delta }}{n}_{2}\equiv {\rm{\Delta }}{n}_{FLC}$), and *d*_*i*_ is the thickness of the phase retarder. From Eqs (1) and (2), four possible Jones matrices (M_*FLC*_) can be written in Eq. (3) after considering two phase retarders.

3 $${{M}_{FLC}}{({{\rm{\Gamma }}}_{1},{{\rm{\Gamma }}}_{2})} = \left\{\begin{array}{ll} {\frac{1}{2}}{\left[\begin{array}{cc}1+{e}^{j({{\rm{\Gamma }}}_{2}+{{\rm{\Gamma }}}_{1})} & 1-{e}^{j({{\rm{\Gamma }}}_{2}+{{\rm{\Gamma }}}_{1})}\\ 1-{e}^{j({{\rm{\Gamma }}}_{2}+{{\rm{\Gamma }}}_{1})} & 1+{e}^{j({{\rm{\Gamma }}}_{2}+{{\rm{\Gamma }}}_{1})}\end{array}\right]}   & {{\theta }_{1}}={{\theta }_{2}}= {{135}^{\circ }} \\{\left[\begin{array}{cc}1 & 0\\ 0 & {e}^{j({{\rm{\Gamma }}}_{2}+{{\rm{\Gamma }}}_{1})}\end{array}\right]} & {{\theta }_{1}}=   {{\theta }_{2}}= {{90}^{\circ }}\\{\frac{1}{2}\left[\begin{array}{cc}1+{e}^{j{{\rm{\Gamma }}}_{1}} & 1-{e}^{j{{\rm{\Gamma }}}_{1}}\\{e}^{j{{\rm{\Gamma }}}_{2}}(1-{e}^{j{{\rm{\Gamma }}}_{1}}) & {e}^{j{{\rm{\Gamma }}}_{2}}(1+{e}^{j{{\rm{\Gamma }}}_{1}})\end{array}\right]} & {{\theta }_{1}} = {{135}^{\circ }},\,{{\theta }_{2}} =  {{90}^{\circ }}\\{\frac{1}{2}\left[\begin{array}{cc}1+{e}^{j{{\rm{\Gamma }}}_{2}} & {e}^{j{{\rm{\Gamma }}}_{1}}(1-{e}^{j{{\rm{\Gamma }}}_{2}})\\ 1-{e}^{j{{\rm{\Gamma }}}_{2}} & {e}^{j{{\rm{\Gamma }}}_{1}}(1+{e}^{j{{\rm{\Gamma }}}_{2}})\end{array}\right]} & {{\theta }_{1}}={{90}^{\circ }},\,{{\theta }_{2}}={{135}^{\circ }}\end{array}\right.$$

To further narrow the bandwidth, a Lyot-Ohman filter is adopted as a bandwidth suppressor^35^. The slow axis of phase retarder 3 is 135 degrees with respect to the x-axis (*θ*_3_ = 135°). The Jones matrix of the bandwidth suppressor (*M*_*BS*_) is expressed as:

4 $${M}_{BS}=\frac{1}{2}\cdot [\begin{array}{cc}0 & 0\\ 0 & 1\end{array}]\cdot [\begin{array}{cc}1+{e}^{j{{\rm{\Gamma }}}_{LC}} & 1-{e}^{j{{\rm{\Gamma }}}_{LC}}\\ 1-{e}^{j{{\rm{\Gamma }}}_{LC}} & 1+{e}^{j{{\rm{\Gamma }}}_{LC}}\end{array}]$$

where Γ_*LC*_ is the phase retardation of phase retarder 3. The function of the lens is to convert the wavefront of the incident light. According to the thin lens approximation, the complex amplitude transmittance function of a lens is^45^ ${e}^{-jk\frac{{r}^{2}}{2f}}$, where *k* is the wave number, *f* is the focal length of a lens, and *r* is $\sqrt{{x}^{2}+{y}^{2}}$. The function of lens L_1_ and lens L_2_ is light collimation. One assumes that the wavefront remains a plane wave and is not altered by lenses L_1_ and L_2_ before incidence onto lens L_3_. As shown in Fig. 2(b), the wavefront of the plane wave is modulated by both of lens L_3_ and the LC lens. The plane wave turns out a paraboloid wave of Gaussian beam centered at point O. When lens L_3_ and the LC lens are placed closed enough, the complex transmittance function for lens L_3_, the LC lens and the transverse distance z is:

5 $${t}_{L3-LC}(z)=\frac{1}{({f}_{e}+d^{\prime\prime} )-z}\cdot {e}^{-jk\frac{{r}^{2}}{2({f}_{e}+d^{\prime\prime} )}}$$

6 $$r=\sqrt{{x}^{2}+{y}^{2}+{[({f}_{e}+d^{\prime\prime} )-z]}^{2}}$$

7 $${f}_{e}=\frac{{f}_{3}\cdot {f}_{LC}}{{f}_{3}+{f}_{LC}}$$

where *f*_3_ and *f*_*LC*_ are the focal lengths of lens L_3_ and the LC lens, respectively. We can apply the output beam in Fig. 2(a) to a target (e.g., skin) located at a distance *d’* away from the LC lens, as shown in Fig. 2(b). Then, according to Eq. (5), the amplitude is varied by ${A}_{0}=({f}_{e}+d^{\prime\prime} )/({f}_{e}+d^{\prime\prime} -d^{\prime} )$. At location *z* = *f*_*e*_, the beam has a finite size instead of a single point due to the diffraction limit. We assume that an unpolarized light with an electric field amplitude of *E*_0_ impinges onto the optical system. From Eqs (3), (4), and (5) and the Jones matrix of the polarizer, the Jones matrix (*M*_*total*_) for the output beam in Fig. 2(a) is expressed as:

8 $$\begin{array}{l}{M}_{total}({{\rm{\Gamma }}}_{1},{{\rm{\Gamma }}}_{2},{{\rm{\Gamma }}}_{LC})\\ \begin{array}{ll}\quad = & {A}_{0}\cdot {e}^{-jk{d}^{\text{'}}}\cdot {e}^{-jk\frac{{r}^{2}}{2({f}_{e}+d^{\prime\prime} )}}\cdot [\begin{array}{cc}0 & 0\\ 0 & 1\end{array}]\frac{1}{2}[\begin{array}{cc}1+{e}^{j{{\rm{\Gamma }}}_{LC}} & 1-{e}^{j{{\rm{\Gamma }}}_{LC}}\\ 1-{e}^{j{{\rm{\Gamma }}}_{LC}} & 1+{e}^{j{{\rm{\Gamma }}}_{LC}}\end{array}]\\  & \cdot \frac{1}{2}[\begin{array}{cc}0 & 0\\ 0 & 1\end{array}]{M}_{FLC}({{\rm{\Gamma }}}_{1},{{\rm{\Gamma }}}_{2})[\begin{array}{cc}0 & 0\\ 0 & 1\end{array}]\\ \quad = & \{\begin{array}{l}\frac{{A}_{0}}{4}{e}^{-jkd^{\prime} }{e}^{-jk\frac{{r}^{2}}{2({f}_{e}+d^{\prime\prime} )}}[\begin{array}{cc}0 & 0\\ 0 & (1+{e}^{j{{\rm{\Gamma }}}_{LC}})\cdot (1+{e}^{j({{\rm{\Gamma }}}_{2}+{{\rm{\Gamma }}}_{1})})\end{array}],when\,{\theta }_{1}={\theta }_{2}=135^\circ \,\\ \frac{{A}_{0}}{2}{e}^{-jkd^{\prime} }{e}^{-jk\frac{{r}^{2}}{2({f}_{e}+d^{\prime\prime} )}}[\begin{array}{cc}0 & 0\\ 0 & (1+{e}^{j{{\rm{\Gamma }}}_{LC}})\cdot ({e}^{j({{\rm{\Gamma }}}_{2}+{{\rm{\Gamma }}}_{1})})\end{array}],when\,{\theta }_{1}={\theta }_{2}=90^\circ \,\\ \frac{{A}_{0}}{4}{e}^{-jkd^{\prime} }{e}^{-jk\frac{{r}^{2}}{2({f}_{e}+d^{\prime\prime} )}}[\begin{array}{cc}0 & 0\\ 0 & (1+{e}^{j{{\rm{\Gamma }}}_{LC}})\cdot (1+{e}^{j{{\rm{\Gamma }}}_{1}}){e}^{j{{\rm{\Gamma }}}_{2}}\end{array}],when\,{\theta }_{1}=135^\circ ,\,{\theta }_{2}=90^\circ \\ \frac{{A}_{0}}{4}{e}^{-jkd^{\prime} }{e}^{-jk\frac{{r}^{2}}{2({f}_{e}+d^{\prime\prime} )}}[\begin{array}{cc}0 & 0\\ 0 & (1+{e}^{j{{\rm{\Gamma }}}_{LC}})\cdot (1+{e}^{j{{\rm{\Gamma }}}_{2}}){e}^{j{{\rm{\Gamma }}}_{1}}\end{array}],when\,{\theta }_{1}=90^\circ ,\,{\theta }_{2}=135^\circ \end{array}\end{array}\end{array}$$

From Eq. (5), the field amplitude (*E*) and the transmittance ($T={|E|}^{2}/{|{E}_{0}|}^{2}$) are given by Eqs (9) and (10):

9 $$E=\frac{{A}_{0}}{2\sqrt{2}}\cdot \sqrt{(1+cos{{\rm{\Gamma }}}_{eff})(1+cos{{\rm{\Gamma }}}_{LC})}\cdot {E}_{0}$$

10 $$T=\frac{1}{8}(1+cos{{\rm{\Gamma }}}_{eff})\cdot (1+cos{{\rm{\Gamma }}}_{LC})\cdot {(\frac{{f}_{e}+d^{\prime\prime} }{{f}_{e}+d^{\prime\prime} +d^{\prime} })}^{2}$$

where Γ_*eff*_ is the effective phase retardation and satisfies Eq. (11).

11 $${{\rm{\Gamma }}}_{eff}({{\rm{\Gamma }}}_{1},{{\rm{\Gamma }}}_{2})=\left\{\begin{array}{cc}{{\rm{\Gamma }}}_{1}+{{\rm{\Gamma }}}_{2} & {\theta }_{1}={\theta }_{2}={135}^{\circ }\\ 0 & {\theta }_{1}={\theta }_{2}={90}^{\circ }\\ {{\rm{\Gamma }}}_{1} & {\theta }_{1}={135}^{\circ },\,{\theta }_{2}={90}^{\circ }\\ {{\rm{\Gamma }}}_{2} & {\theta }_{1}={90}^{\circ },\,{\theta }_{2}={135}^{\circ }\end{array}\right.$$

To design the maximal transmittance in Eq. (10) for three center wavelengths of 450 nm, 550 nm and 650 nm, the phase retardation of phase retarder 1 and phase retarder 2 should satisfy Eq. (12).

12 $${{\rm{\Gamma }}}_{i}=\frac{2\pi }{\lambda }\cdot {{\rm{\Delta }}n}_{i}\cdot {d}_{i}=2\pi \cdot {N}_{i}\,,\,{N}_{i}\in int\,,\,and\,i=1,2$$

The birefringence of both phase retarders is 0.17 in the experiments. According to Eq. (11), the thickness should be 2.65 *N*_1_, 3.23 *N*_2_, and 3.82 *N*_3_ for λ = 450 nm, 550 nm, and 650 nm, respectively. *N*_1_, *N*_2_, and *N*_3_ are three integers. We set *d*_1_ = 2.65 μm for phase retarder 1 and *d*_2_ = 3.82 μm for phase retarder 2. This means that blue light (λ = 450 nm) has a maximal transmittance as Γ_*eff*_ = Γ_1_ (blue mode) and red light (λ = 650 nm) has a maximal transmittance as Γ_*eff*_  = Γ_2_ (red mode). When Γ_*eff*_ = Γ_1_ + Γ_2_, green light (λ = 550 nm) has a maximal transmittance (green mode) according to Eq. (12). As a result, three wavelength can be selected by controlling phase retardations of the two phase retarders. In Fig. 2(a), after the white light passes through the electrically tunable color filter as *θ*_1_ = *θ*_2_ = 90° (defined as the off-state for both of the phase retarders), the spectrum does not change because there is no phase retardation for the y-linearly polarized light. However, light passing through the electrically tunable color filter turns out to be green as *θ*_1_ = *θ*_2_ = 135° (defined as the on-state for both of the phase retarders) because the green polarized light reaches maximal transmittance. Similarly, when one phase retarder is in the on-state and the other is in the off-state, the light turns out to be blue or red depending on Γ_*eff*_ = Γ_1_ or Γ_2_. In Eq. (9), the term $\sqrt{1+cos{{\rm{\Gamma }}}_{LC}}$ reduces the bandwidth for the three wavelengths.

From Eq. (5) and Fig. 2(b), the beam size is ${\rm{D}}\times ({f}_{e}(V)+d^{\prime\prime} -d^{\prime} )/({f}_{e}(V)+d^{\prime\prime} )$, where D is the beam size before entering the lens L_3_ and ${f}_{e}(V)=({f}_{3}\times {f}_{LC}(V))/({f}_{3}+{f}_{LC}(V))$ is the equivalent focal length of the combination of L_3_ and LC lens. The focal length of the LC lens is voltage (V)-dependent and satisfies the relation^37^:

13 $${f}_{LC}(V)=\frac{{r}_{0}^{2}}{2\times \delta n(V)\times l}$$

where *r*_0_ is the aperture size of the LC lens, *l* is the thickness of the LC lens, and $\delta n$ is the refractive index difference between the center and border of the LC lens. The focal length of the LC lens can be switched continuously from a negative value to a positive value by the applied voltage. As a result, the beam size of the output shown in Fig. 2(a) is electrically adjustable. Therefore, we demonstrate that the light wavelengths as well as the beam size are both electrically switchable in the optical system illustrated in Fig. 2(a). By adjusting the period for light exposure, the fluence or energy intensity is also switchable.

**Figure 3:**
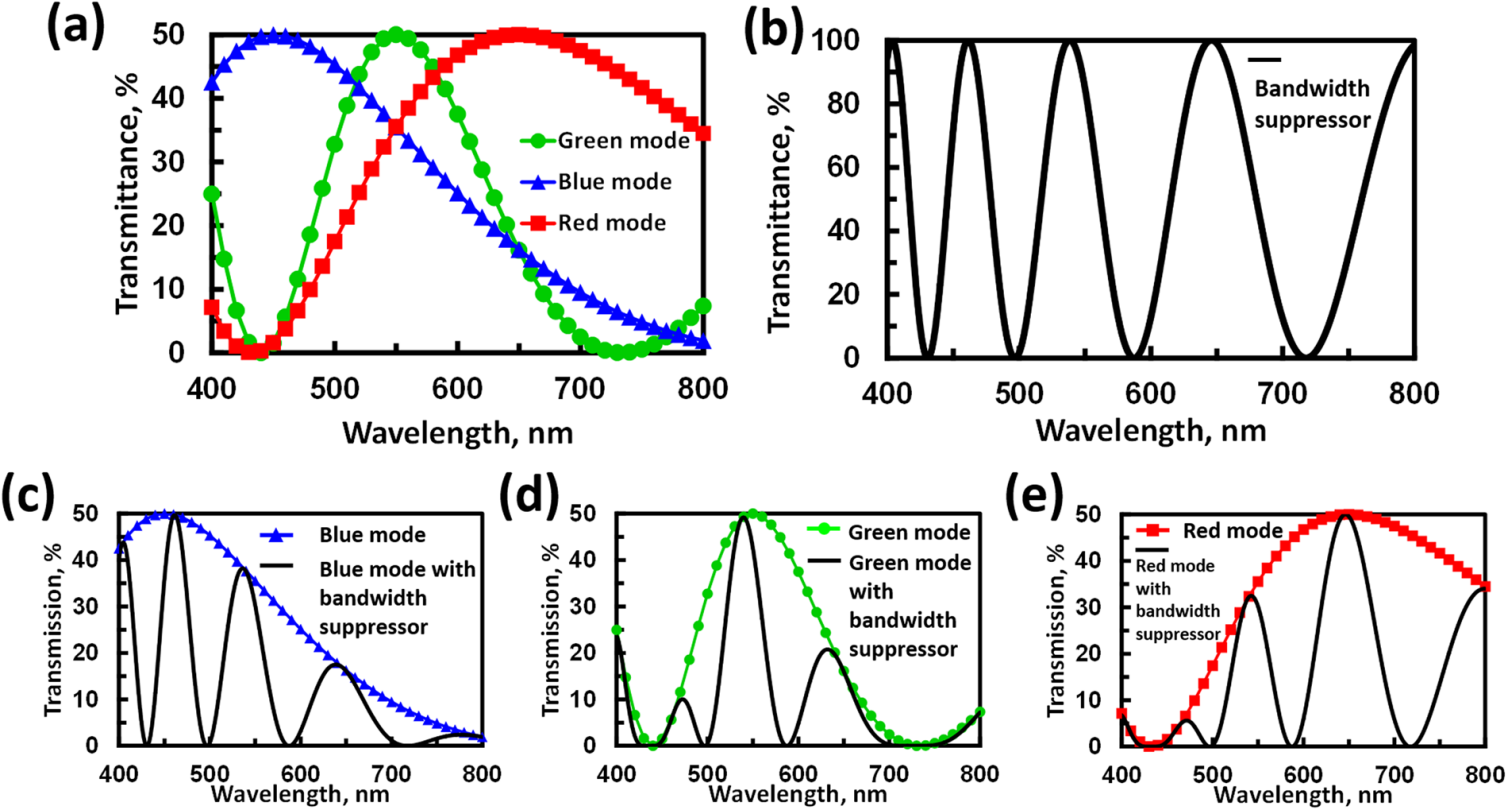
(**a**) Calculated transmissive spectrum of the optical system for the electrically tunable color filter only. (**b**) Calculated transmissive spectrum for the bandwidth suppressor only. By combining (**a**) and (**b**), (**c**), (**d**), and (**e**) Illustrate the transmissive spectra for the blue mode, green mode and red mode with the bandwidth suppressor, respectively. The parameters used are: Δn = 0.17, d_1_ = 2.65 μm and d_2_ = 3.82 μm, Γ_LC_ is set as 6.46π/λ radians.

Figure 3(a) shows the calculated transmittance of $(1+cos{{\rm{\Gamma }}}_{eff})/4$ as a function of wavelength for the blue, green, and red modes, which represents the transmittance of the electrically tunable color filter only (Fig. 2(a)). The data show that three center wavelengths are indeed located at λ = 450 nm, 550 nm, and 650 nm. The full width at half maximum (FWHM) is 239.4 nm for the blue mode, 245.4 nm for the red mode, and 131.9 nm for the green mode. Figure 3(b) illustrates the transmittance of $1+cos{{\rm{\Gamma }}}_{LC}$ as a function of wavelength. The phase retardation of Γ_*LC*_ is set as 6.46π/λ radians to satisfy Eq. (12) as a maximum wavelength of λ = 646 nm, 538 nm, and 461 nm. When we combine the tunable color filter with the bandwidth suppressor, the transmissions of $(1+cos{{\rm{\Gamma }}}_{eff})\cdot (1+cos{{\rm{\Gamma }}}_{LC})/4$ vs. wavelength for the blue, green, and red modes are illustrated in Fig. 3(c)–(e), respectively. In Fig. 3–(e), the FWHM at the center wavelengths of 450 nm, 550 nm, and 650 nm are 32.4 nm, 42.6 nm, and 63.6 nm, respectively. The values for the FWHM of the three modes are all less than 100 nm under the assistance of the bandwidth suppressor. By adjusting the Γ_*LC*_ in Eq. (10), the FWHM can be reduced further. The side lobes of the transmittance spectrum can be further reduced by adding more bandwidth suppressors with designed parameters.

### Sample preparation

**Figure 4:**
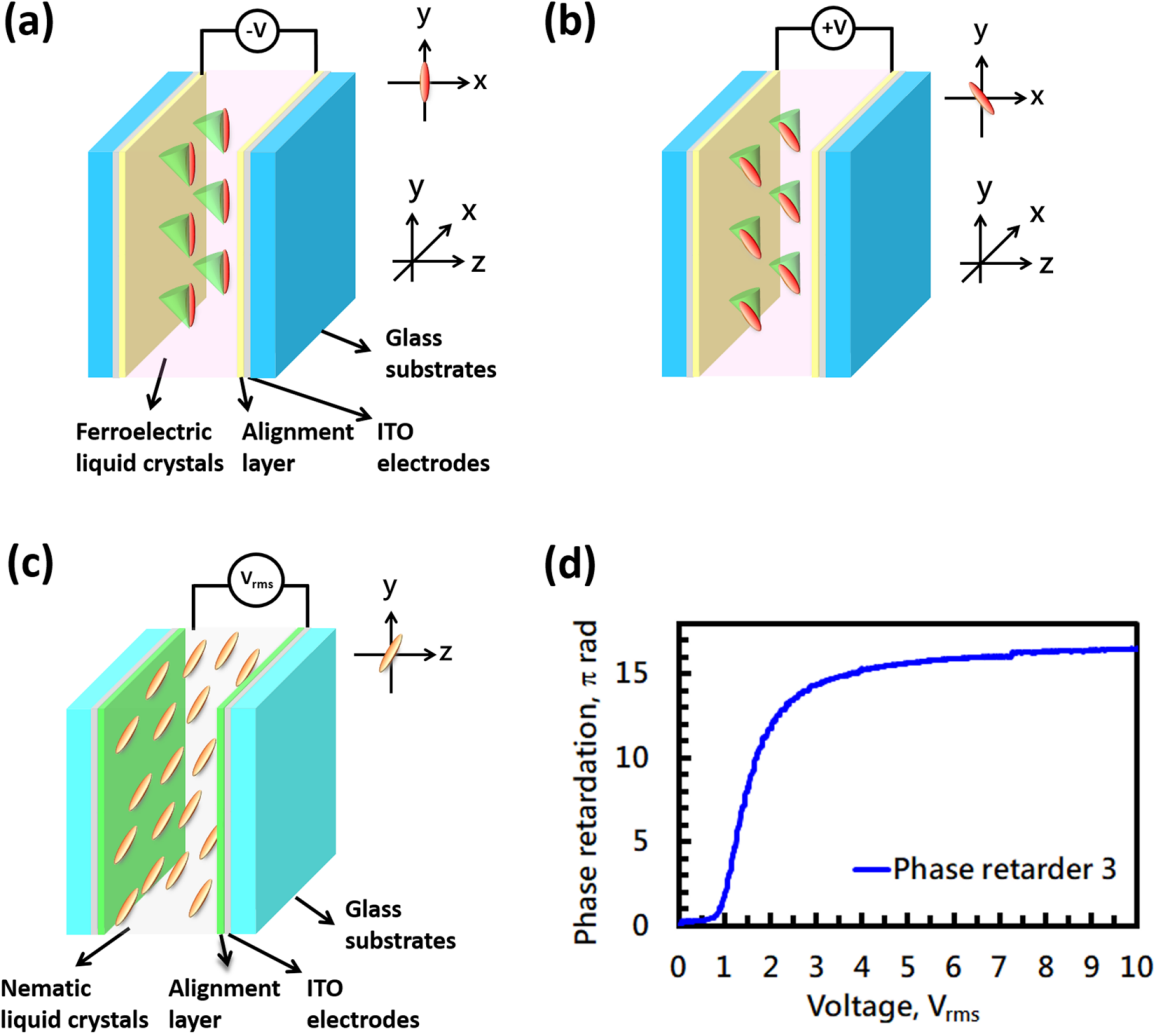
The structure of phase retarder 1 and phase retarder 2 using an FLC, (**a**) when the FLC phase retarder is subject to an applied voltage of −10 V and (**b**) +10 V. (**c**) The structure of phase retarder 3 using nematic LC when the voltage is switched off. (**d**) The phase retardation of phase retarder 3 as a function of applied voltage.

Ferroelectric liquid crystal (FLC) was used in both phase retarder 1 and phase retarder 2. FLC material (Felix-017/000, Δn = 0.17) was sandwiched between two ITO glass substrates coated with polyimide layers (Mesostate LCD industries, Taiwan) and mechanically rubbed in anti-parallel directions. The cell gaps for the two FLC cells were 2.8 μm (phase retarder 1) and 3.8 μm (phase retarder 2). The LC material used for phase retarder 3 was nematic LC (E7, Δn = 0.21, λ = 650 nm). The nematic LC was also sandwiched between two ITO glass substrates coated with polyimide layers (Mesostate LCD industries) and mechanically rubbed in anti-parallel directions. The cell gap was 25.6 μm. The structures of the FLC phase retarders (phase retarder 1 and 2) and phase retarder 3 are shown in Fig. 4(a)–(c). The initial alignments for the FLC phase retarders (rubbing directions) were either 90° or 135° with respect to the x-axis. When we applied −10 V to the FLC phase retarder, the LC directors were switched to 90° with respect to the x-axis, as shown in Fig. 4(a). When we applied +10 V to the FLC phase retarder, the LC directors were switched to 135° with respect to the x-axis, as shown in Fig. 4(b). Two typical bistable states for the FLC phase retarders were designed for wavelength selection. The transmittance of the FLC phase retarder under crossed-polarizers as a function of applied voltage was tested in a previous study^44^. The phase retardation for phase retarder 3 as a function of applied voltage is shown in Fig. 4 (d). To meet the desired optical path difference (~3.23 μm), the LC cell for the bandwidth suppressor was operated at an applied voltage of 1.75 V_rms_ (*f* = 1 kHz). The reason why we used FLC materials is the fast response time of sub-milliseconds. Actually, we can also utilize nematic liquid crystals in phase retarders 1 and 2.

**Figure 5:**
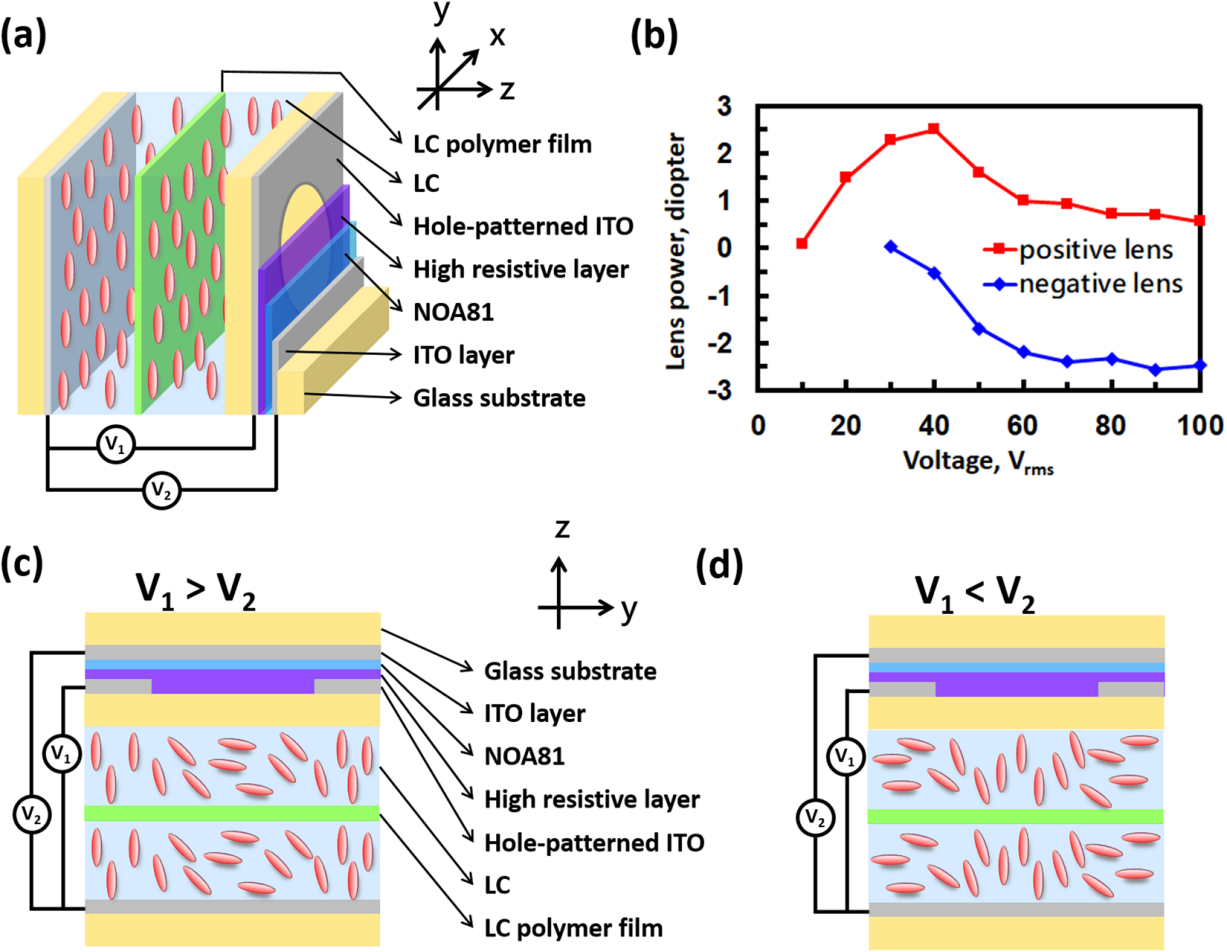
(**a**) The structure of the LC lens. (**b**) The voltage-dependent lens power of the LC lens. Red squares represent the lens power at different V_1_ for V_2_ = 10 V_rms_ and f = 4.2 kHz. Blue diamonds represent the lens power at different V_2_ for V_1_ = 30 V_rms_ and f = 800 Hz. The LC lens is (**c**) a positive lens when V_1_ > V_2_ and (**d**) a negative lens when V_2_ > V_1_.

For the LC lens, we adopted an LC lens in double-layered structure with a hole-patterned electrode and two flat electrodes^37^. The glass substrates were 0.4 mm in thickness. High resistive layer was spin coated onto the hole-patterned electrode. The NOA81 served as insulating layer with a thickness of 25 μm. Instead of being aligned othogonally, the two LC layers (nematic liquid crystal, LCMatter, LCM-1790) were aligned in parallel direction, because we need a large polarization-dependent optical phase here. The detailed structure for the LC lens is shown in Fig. 5(a). The cross-sections of the LC lens at voltage-on are illustrated in Fig. 5(c) and (d). A hole-patterned electrode and two flat electrodes (gray-colored) control the inhomogeneous electric fields distributed across the LC layers. Lens powers are controlled by the two electric fields V_1_ and V_2_. While at the applied voltages V_1_ > V_2_, LC molecules in the center of the LC lens are more parallel than the molecules near the edge of the aperture. As a result, light travels faster near the edge of the aperture than in the center. An incident plane wave is then converted to a converged paraboloidal wave. The lens power of the LC lens is positive. In contrast, the LC lens is a negative lens for V_2_ > V_1_. The thickness of the two LC layers and the LC polymer film was 50 μm. The aperture size was 10 mm. The voltage-dependent lens power was measured based on a Shack-Hartmann wavefront sensor (Thorlab, WFS-150-7AR). Figure 5(b) shows the measured lens power as a function of V_1_ and V_2_. The measured lens power for the LC lens ranges from +2 Diopter to −2.5 Diopter (i.e., focal length from −40 cm to +50 cm).

## Results and Discussion

### Transmission spectra of the retarders

**Figure 6:**
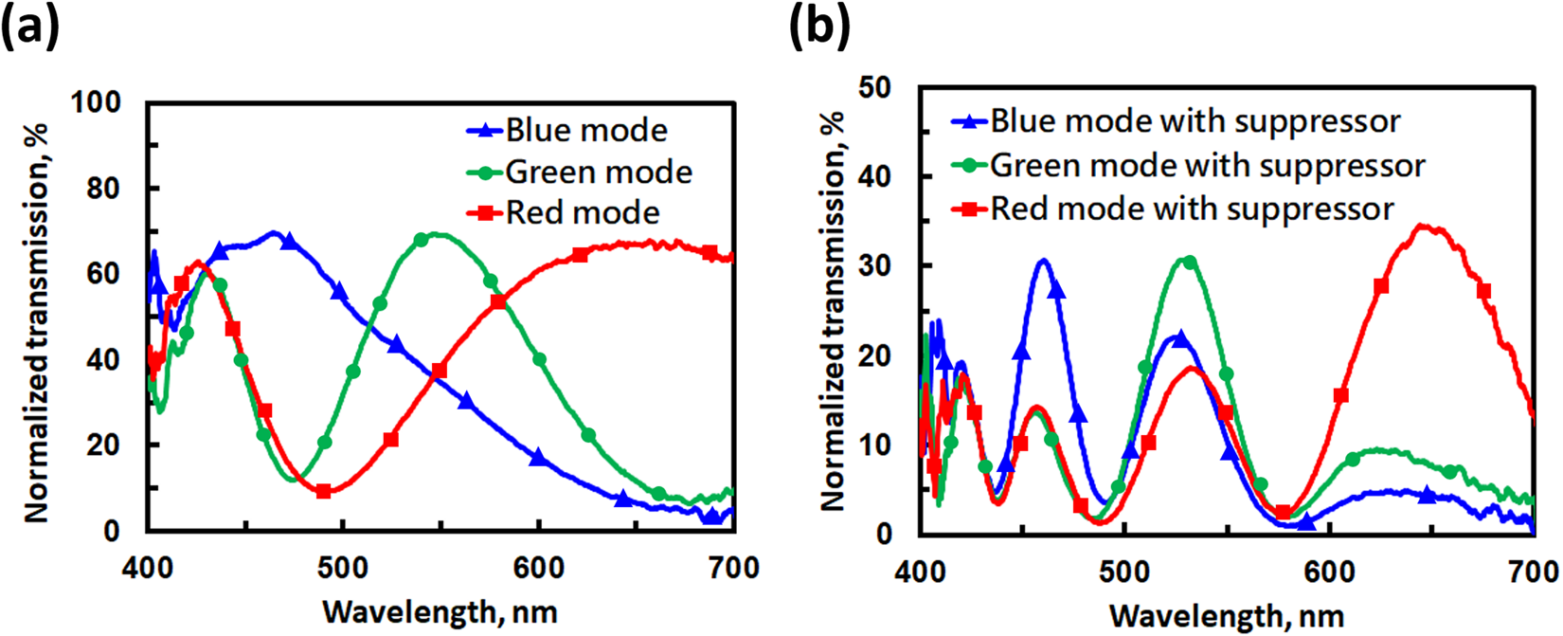
Experimental results showing the normalized transmission spectra for (**a**) the electrically tunable color filter without a bandwidth suppressor and (**b**) the electrically tunable color filter with a bandwidth suppressor.

After fabrication of the two FLC phase retarder cells, namely, the 1^st^ FLC phase retarder (phase retarder 1) with a measured cell gap of 2.8 μm and the 2^nd^ FLC phase retarder (phase retarder 2) with a cell gap of 3.8 μm, we measured transmissive spectra for the tunable color filter. An LED light (TouchBrigh TB-X3-RCPI) was used as a white light source. Two solid lenses and a pinhole were used to collimate the light. To construct the tunable color filter (Fig. 1(a)), two FLC samples were placed between two polarizers with transmissive axes parallel to each other (y-axis in Fig. 1(a)). A spectrometer (USB-2000, Ocean Optics) was used to measure the spectra. The measured spectra for the tunable color filter (no bandwidth suppressor) are shown in Fig. 6(a). The data were normalized to the spectrum of the light source through two polarizers with parallel transmissive axes. The lines with blue triangles, red squares, and green dots represent the blue mode (i.e., 1^st^ FLC is on, 2^nd^ FLC is off), red mode (i.e., 1^st^ FLC is off, 2^nd^ FLC is on), and green mode (i.e., both of FLC cells are on), respectively. In Fig. 6(a), the center wavelengths of the three modes are located at 450 nm (blue mode), 550 nm (green mode), and 650 nm (red mode). The FWHM values are 171.3 nm at a center wavelength of 450 nm, 92.3 nm at a center wavelength of 550 nm, and 213.1 nm at a center wavelength of 650 nm. Figure 6(b) shows the spectra after adding the bandwidth suppressor to the tunable color filter. As one can see from the data, the FWHM near the three center wavelengths is suppressed. After the bandwidth suppressor, the FWHM values are 28.3 nm at λ = 450 nm, 44.6 nm at λ = 550 nm, and 84.9 nm at λ = 650 nm. Comparing Fig. 6(a) to (b), the normalized transmission in Fig. 6(b) is lower than that in Fig. 6(a) because of the polarizer and multiple reflections between the interfaces. In addition, the side lobes appear outside the center wavelengths, which could be reduced by enlarging the optical path differences for the phase retarders; thus, an even narrower FWHM is achievable.

**Figure 7:**
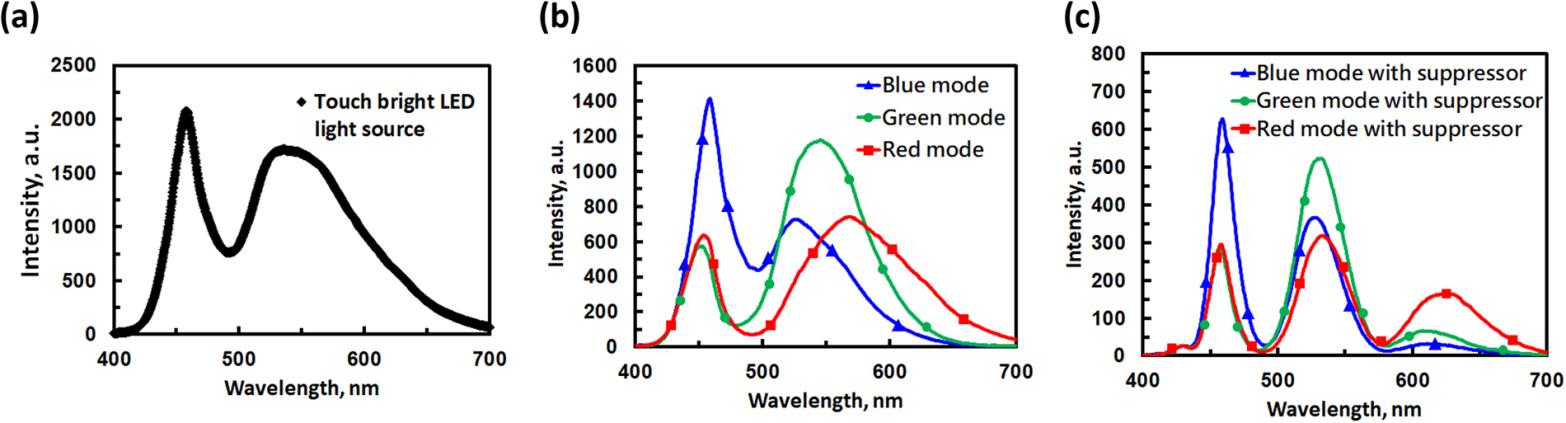
Light intensity spectra for (**a**) LED light source, (**b**) electrically tunable color filter and (**c**) electrically tunable color filter with bandwidth suppressor.

The spectrum of the LED light source could also affect the spectra of the design. The spectrum of the LED light source used is shown in Fig. 7(a). The spectra for the three modes measured after light travels through the electrically tunable color filter are shown in Fig. 7(b). The spectra for three modes measured after light propagation through both the electrically tunable color filter and bandwidth compressor are shown in Fig. 7(c). In Fig. 7(b) and (c), the bandwidths (FWHM) change from 31.5 to 19.5 nm for λ = 458.5 nm (blue mode), 73.8 to 37.3 nm for λ = 545.7 nm (green mode), and 102.9 to 41.4 nm for λ = 568 nm (red mode). From Fig. 7(c), the peak intensity for the red mode at λ = 650 nm is low, with two side lobes observed in the blue and green regions. This is because the spectrum of the LED light source in Fig. 7(a) shows two main peaks at λ = 458 nm and 536 nm. To increase the intensity of the red light, we can use a light source with a more uniform spectrum, such as a halogen lamp or a xenon lamp. In other words, the purity of RGB color in Fig. 7(c) could be improved by adjusting the spectrum of light source and adopting more bandwidth suppressors to remove the side lobes.

### Tunable irradiance by a liquid crystal lens

**Figure 8:**
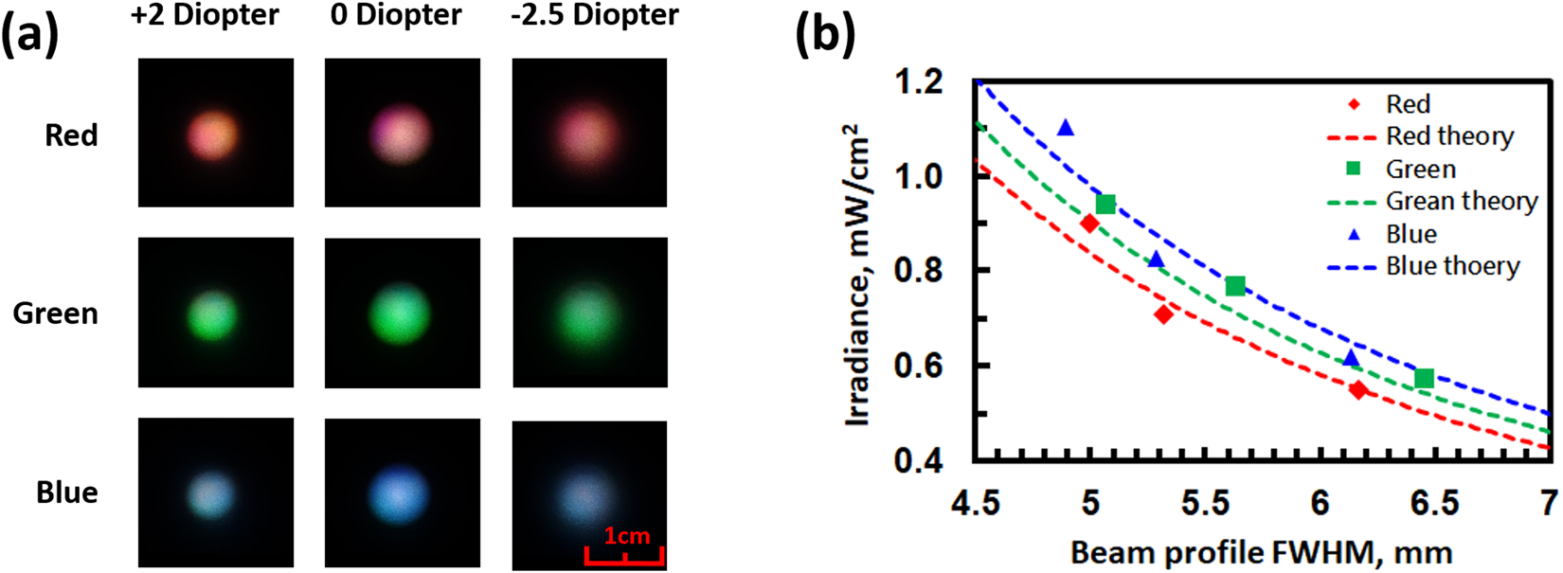
(**a**) Beam spots at different lens power for the LC lens at different modes. (**b**) Irradiance as a function of beam diameter at different modes. The theoretical curves are obtained based on Eqs (5) and (10).

In addition to the tunable color filter and the bandwidth compressor, an LC lens in the system shown in Fig. 2(a) provides the capability of tunable irradiance. The function of the lens L_3_ is to adjust the beam size ~10 mm incident to the LC lens. By means of changing the beam size at the exposure area by tunable lens power, the irradiance in units of Watt/m^2^ can be adjusted. To measure the change in exposure area, we placed a diffusor at a distance of 7 cm away from the LC lens and recorded images taken by a camera (Sony RX100-m3) when the LC lens was operated at different lens powers and the system operated at three modes. The recorded images are shown in Fig. 8(a). The images in the first row of Fig. 8(a) show the difference in beam size at different lens powers for the LC lens at the red mode. The second and third rows show images for the green mode and blue mode. To further analyze the spot profile, images were first averaged by software (ImageJ) before fitting the brightness distribution at the x-cross-section of each spot by a Gaussian function to calculate the FWHM. The FWHM of each spot is slightly enlarged in diameter from 5 mm to 6.5 mm when the lens power changes from +2 Diopter to −2.5 Diopter. We also plot the irradiance as a function of beam size in Fig. 8(b). The tunable range of the irradiance for all three wavelengths is approximately 0.6 to 1 mW/cm^2^. The irradiance decreases with increasing beam size, which results from the lens power of the LC lens. From experiments, d″ in Fig. 2(b) is approximately 7 cm. We can calculate the theoretical relation between the beam size and irradiance according to Eq. (5). This is shown by the dashed lines in Fig. 8(b). As a result, the optical system we proposed in Fig. 2(a) not only is capable of wavelength selection but also has the ability to adjust irradiance. By multiplying the irradiance by time, one can tune the energy density (or fluence). For practical application, the energy density (or fluence) in our system can reach 2.16~3.60 J/cm^2^ for a treatment time of 60 min. The treatment time is still too long for a portable light therapy device. This problem can be solved by decreasing the power loss of the optical system in terms of removal of multiple reflections. If the irradiance of the output light can reach a range of 2.4~4 mW/cm^2^, the treatment time can be reduced to approximately 15 minutes.

According to the literature^46^, the theoretical spot size for incident collimated laser light is 3.4 mm, 1.6 mm, and 0.2 mm for an LC lens power of −2.5D, 0D, and +2D, respectively. We assume that the spot is located at a distance 7 cm away from the LC lens. Compared to experiments, the spot size is quite different. This is because the light source is an LED rather than a collimated laser beam. The LED light that propagates towards the lens L_3_ is not a plane wave. This could be improved by adopting a collimated white laser light or LED with better collimation for enlargement of the irradiance. In addition, as the number of phase retarders in the tunable color filter increases, the selection of wavelengths increases. As the number of phase retarders in the bandwidth suppressor increases, the bandwidth decreases, and the side lobes diminish. The spectrum of the light source can be adjusted to be more uniform for better tunability of the irradiance at different wavelengths. Considering that the exposure time is controlled by a light shutter, the optical system we have proposed in this paper exhibits electrical tunability of wavelength as well as optical density or the so-called dose. As to the efficiency of light in this system, it is around 15%, which mainly originates from the polarizer and color selection of the phase retarders. Even though the efficiency is not high, the fluence of the output light is still high enough for biophotomodulation.

## Conclusion

We demonstrate an optical system based on liquid crystal photonic devices for photobiomodulation or low-level light therapy. By properly designing LC phase retarders and LC lenses, the optical system shows electrical tunability of the wavelength and energy density for an LED white light source. Two FLC phase retarders are used as a tunable color filter for three wavelengths (red, blue and green), an LC phase retarder using a nematic LC is used as a bandwidth suppressor, and a liquid crystal lens is used for adjusting the beam size. The bandwidth of the peak wavelength is suppressed to less than 100 nm. The energy density is manipulated from 0.6 to 1 mW/cm^2^ by tuning the exposure area size using an LC lens. The concept described in this paper can be extended to other photonic devices as long as the photonic devices have the capability for tunable phase retardations and wavefront conversion (i.e., lens). The photonic devices can be further designed and tailored to the needs of photobiomodulation. We hope this study can facilitate clinical progress for photobiomodulation.
